# Anti-microRNAs as Novel Therapeutic Agents in the Clinical Management of Alzheimer's Disease

**DOI:** 10.3389/fnins.2016.00059

**Published:** 2016-02-25

**Authors:** Yuhai Zhao, Peter N. Alexandrov, Walter J. Lukiw

**Affiliations:** ^1^LSU Neuroscience Center, Louisiana State University Health Science CenterNew Orleans, LA, USA; ^2^Department of Cell Biology and Anatomy, Louisiana State University Health Science CenterNew Orleans, LA, USA; ^3^Russian Academy of Medical SciencesMoscow, Russia; ^4^Department of Ophthalmology, LSU Neuroscience Center, Louisiana State University Health Science CenterNew Orleans, LA, USA; ^5^Department Neurology, LSU Neuroscience Center, Louisiana State University Health Science CenterNew Orleans, LA, USA

**Keywords:** aging, Alzheimer's disease, anti-microRNA (AM), human genetic heterogeneity, inflammation, microRNA (miRNA), NF-kB, non-coding RNA

## Abstract

**Overview-** One hundred and ten years since its first description Alzheimer's disease (AD) still retains its prominent status: (i) as the industrialized world's number one cause of age-related intellectual impairment and cognitive decline; (ii) as this country's most rapidly expanding socioeconomic and healthcare concern; and (iii) as an insidious, progressive and lethal neurological disorder of the human central nervous system (CNS) for which there is currently no adequate treatment or cure (Alzheimer, 1991; Alzheimer et al., 1991, 1995) [https://www.alz.org/facts/downloads/facts_figures_2015.pdf (2015)]. The concept of small non-coding RNAs (ncRNAs) as being involved in the etiopathogenesis of AD and age-related human neurodegenerative disease was first proposed about 25 years ago, however it was not until 2007 that specific microRNA (miRNA) abundance, speciation and localization to the hippocampal CA1 region (an anatomical area of the human CNS specifically targeted by the AD process) was shown to strongly associate with AD-type change when compared to age-matched controls (Lukiw et al., 1992; Lukiw, 2007; Schipper et al., 2007; Cogswell et al., 2008; Guerreiro et al., 2012). Currently about 400 reports address the potential link between disruptions in miRNA signaling and the development of various features associated with AD neuropathology (http://www.ncbi.nlm.nih.gov/pubmed/?term=micro+RNA+alzheimer's+disease). In this “*Perspectives*” paper we will highlight some of the most recent literature on anti-miRNA (AM; antagomir) therapeutic strategies and some very recent technological advances in the analysis and characterization of defective miRNA signaling pathways in AD compared to neurologically normal age-matched controls.

## The microRNA (miRNA) mechanism in health and disease—what the numbers are telling us

Firstly, miRNAs are generated within the nucleus by an RNA Polymerase II (RNA Pol II)-mediated transcription mechanism yielding a long “primary” miRNA precursor thousands of nucleotides (nt) in length which are subsequently processed to produce a second miRNA precursor (pre-miRNA) of 60–100 nt. This pre-miRNA next translocates to the cytoplasm where it is further processed by an RNase-III type endoribonuclease and associated ribonuclear proteins to generate a final mature ~22 nt miRNA product (Ambros et al., 2003; Ambros, 2004; Burmistrova et al., 2007; Bartel, 2009; Lukiw, 2012; Jiang et al., 2013; Lukiw et al., 2013; Pogue et al., 2014; Qiu et al., 2014; Denzler and Stoffel, 2015; Fang and Bartel, 2015; Femminella et al., 2015; Karnati et al., 2015; Wu et al., 2015; Zhao et al., 2015; Roth et al., 2016) [http://www.lcsciences.com/applications/transcriptomics/mirna-profiling/mirna/ (2015)]. The initial generation of the long “primary” miRNA, the translocation of miRNA from nucleus to cytoplasm; the “gating” of pre-miRNA precursor through the nuclear pore, the pre-miRNA and the actual mature miRNA ribonucleotide sequence are all potentially important nodes of anti-miRNA- (AM)-based inhibition (Bartel, 2009; Guo et al., 2010; Lukiw, 2012; Denzler and Stoffel, 2015; Fang and Bartel, 2015; Karnati et al., 2015). There are about 2650 mature human miRNAs currently recognized using existing technologies that include, most recently, microfluidic miRNA array and RNA sequencing, and probably not many more novel mature miRNA sequences will be discovered (Burmistrova et al., 2007; Jiang et al., 2013; Lukiw et al., 2013) [http://www.lcsciences.com/applications/transcriptomics/mirna-profiling/mirna/ (2015)]. What is remarkable is the relatively small number of ***abundant*** miRNAs in the human CNS which currently number only about 40–50 (Jiang et al., 2013; Lukiw et al., 2013; Qiu et al., 2014; Femminella et al., 2015; Zhao et al., 2015; Roth et al., 2016) [http://www.lcsciences.com/applications/transcriptomics/mirna-profiling/mirna/ (2015)]. This in itself represents a tremendous evolutionary selection pressure in that only ~1.5% of all known miRNAs have been selected to function in productive gene expression regulation in the brain and CNS (Pogue et al., 2014). For example, miRNA-854, a modulator of the expression of the oligouridylate (oligo-UTP) binding protein 1b (UTPBP1b) has been conserved between *Arabidopsis thaliana* and the brain of *Homo sapiens* for ~1.5 billion years; few nucleic acids, proteins or other molecular markers of any type have been perfectly conserved between plants and animals for such a long period of time (Lukiw, 2012; Pogue et al., 2014; Zhao et al., 2015). Another interesting consideration is that a ~22 nucleotide (nt) single-stranded ncRNA composed of 4 different ribonucleotides—(adenine, guanine, cytosine or uridine; A, G, C, U)—has the potential to form well over 10^13^ possible ~22 oligonucleotide sequence combinations. The fact that there are only about 2.65 × 10^3^ different miRNAs in the entire human body again suggests an extremely high evolutionary selection pressure to utilize only specific ribonucleotide sequences in miRNAs that will yield neuro-biologically useful micro RNA–messenger RNA (miRNA–mRNA) interactions (Ambros et al., 2003; Ambros, 2004; Bartel, 2009; Lukiw, 2012; Denzler and Stoffel, 2015; Fang and Bartel, 2015; Karnati et al., 2015; Wu et al., 2015). Put another way, just about one in ten billion potential miRNA sequences have found a useful purpose in miRNA-mRNA-based gene regulation in human metabolic and developmental physiology, and less than one in two-hundred billion potential miRNA sequences have found a useful purpose in miRNA-mRNA-based gene regulation in the human brain (Bartel, 2009; Lukiw, 2012; Denzler and Stoffel, 2015; Fang and Bartel, 2015). It is an interesting question to ask if these exceptionally unique miRNA sequences in the brain are the common denominator of some CNS gene-regulatory network that we are just beginning to understand. From what we currently know, of the 40–50 abundant miRNAs detected in the “***normally aging”*** control brain and retina only about one-quarter to one-fifth are significantly up-regulated in the AD brain, and this has important implications on the therapeutic design for managing the abundance of that family of pathologically up-regulated miRNAs in the degenerating CNS (Burmistrova et al., 2007; Jiang et al., 2013; Lukiw et al., 2013; Qiu et al., 2014; Femminella et al., 2015; Zhao et al., 2015; Roth et al., 2016) [http://www.lcsciences.com/applications/transcriptomics/mirna-profiling/mirna/ (2015)].

## Pathologically up-regulated miRNAs lead to pathologically down-regulated mRNAs

**Figure 1:**
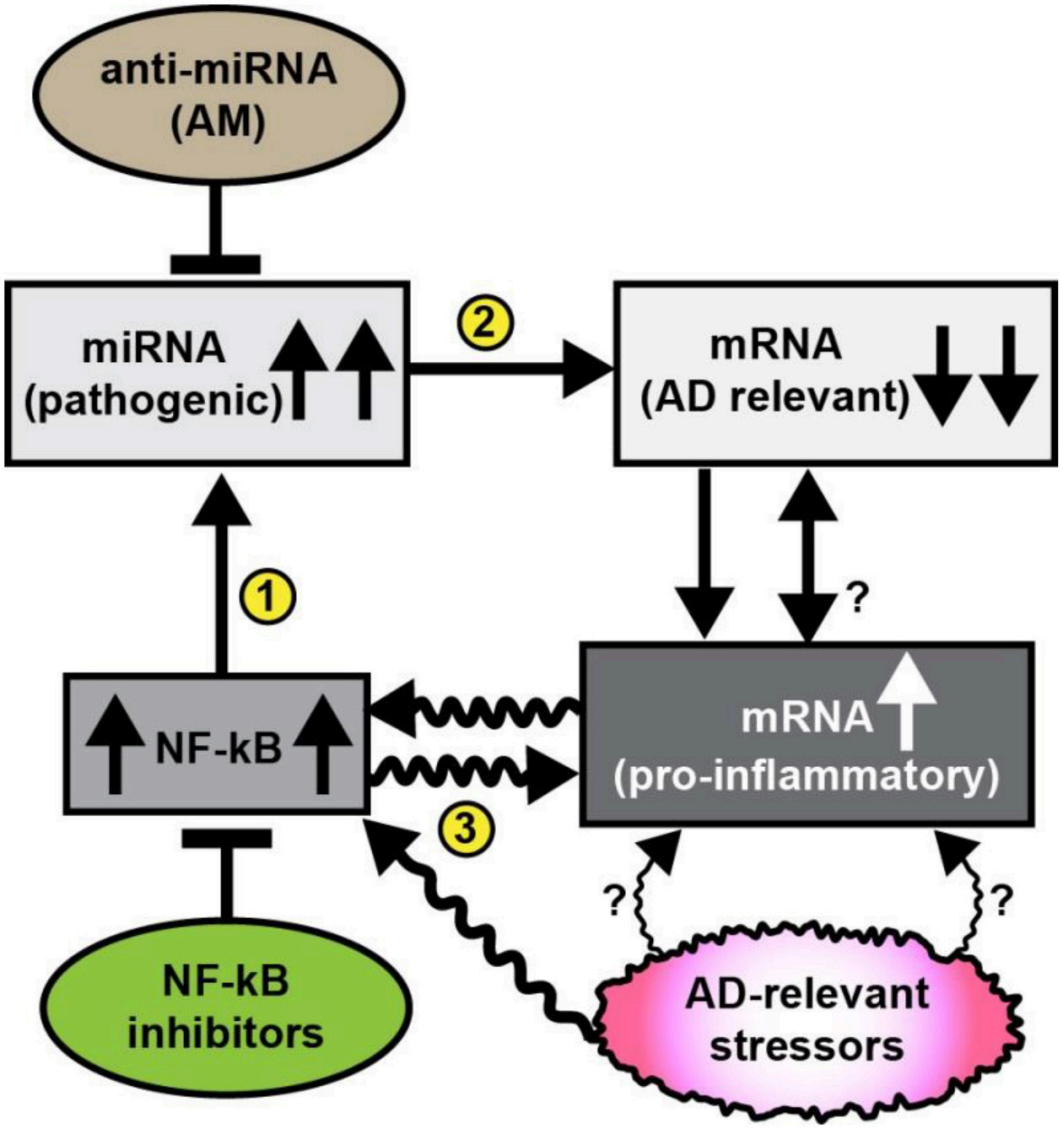
**Schematic diagram illustrating pathogenic signaling circuits in sporadic AD brain and potential therapeutic strategies for intervention. Recent observations suggest the presence of a cyclical and self-reinforcing pathogenic “AD relevant stressor(s)-NF-kB- miRNA-mRNA-gene expression loop” in AD brain; this signaling pathway may be amenable to anti-miRNA (AM) and/or NF-kB inhibitor therapeutic strategies that in theory should help restore homeostatic gene expression to the damaged brain**. Sporadic AD relevant stressors include age, diet (such as high fat-cholesterol intake), environmental, lifestyle, exposure to viruses (such as HCV or HSV-1), environmental toxins (such as aluminum), epilepsy, prions, and other neurological diseases and other factors; each of these AD-relevant stressors are known induce NF-kB signaling (Lukiw et al., 2008; Cui et al., 2010; Lukiw, 2012a,b, 2013a; Hill et al., 2015b; Srinivasan and Lahiri, 2015; Yuan et al., 2015); NF-kB signaling has been observed to be generally up-regulated in AD brain that subsequently up-regulates the transcription from NF-kB sensitive pro-inflammatory miRNA promoters (Lukiw and Bazan, 1998; Lukiw et al., 2008; Cui et al., 2010; Devier et al., 2015; Kaur et al., 2015; Srinivasan and Lahiri, 2015); in turn up-regulated miRNAs generally down-regulate their mRNA targets; for example miRNA-146a, a well characterized pro-inflammatory miRNA contains three active canonical NF-kB recognition features in its promoter (Lukiw et al., 2008; Cui et al., 2010); up-regulated miRNAs may pathologically down-regulate a family of AD relevant mRNAs that are involved in synaptogenesis, neurotrophism, the innate-immune response, inflammation and amyloidogenesis (Loring et al., 2001; Cogswell et al., 2008; Kaur et al., 2015; Srinivasan and Lahiri, 2015). It has been possible, for example, to throttle-up or throttle down miRNA-146a expression in AD primary brain cell culture models using AD-relevant inducers or combinations of AM ribonucleotide sequences and NF-kB inhibitors (Lukiw et al., 2008; Cui et al., 2010; Lukiw, 2012a, 2013a). AD-relevant stressors and NF-kB may also directly induce pro-inflammatory mRNAs that possess NF-kB binding sites in their immediate promoters. While it may be difficult to reduce lifelong exposure to AD relevant stressors it may be possible (i) to specifically inhibit miRNA up-regulation using anti-miRNA (AM) ribonucleotide sequences 100% complementary to up-regulated brain miRNAs, and/or (ii) to directly inhibit NF-kB up-regulation (several hundred NF-kB inhibitors are known). The intrinsic problem with excessive NF-kB inhibition is that many homeostatic cell processes rely on NF-kB signaling so the potential for unwanted off-target effects using NF-kB inhibitors may be both severe and numerous (Lukiw, 2012a,b, 2013a; Yuan et al., 2015). The actions of miRNA signaling may in turn modulate NF-kB signaling. Overall, it may be a better strategy to inhibit miRNAs using highly specific and selective AM sequences to target just a single or a few miRNA-mRNA-mediated signaling pathways, particularly those which are the most significantly up-regulated in AD brain. The combinatorial use of AM strategies and NF-kB inhibitors may turn out to be the most effective in the clinical management of AD, and their stoichiometry may need to be carefully adjusted to meet the needs of individual AD patients (Lukiw et al., 2008; Cui et al., 2010; Lukiw, 2012a,b, 2013a; Srinivasan and Lahiri, 2015; Yuan et al., 2015). The efficient delivery of AM ribonucleotide sequences remains a challenging therapeutic problem (Janssen et al., 2013; Zhang et al., 2013; Brites and Fernandes, 2015; Kang et al., 2015; Properzi et al., 2015; van der Pol et al., 2016; Wang et al., 2015).

While our insights into the neurobiological mechanism and relevance of miRNA signaling continues to evolve, it is now generally accepted that the primary mode of miRNA action is to recognize and bind to specific complementary ribonucleotide sequences in the 3′ untranslated region (3′UTR) of target messenger RNAs (mRNAs), and in doing so, down-regulate their expression (Bartel, 2009; Guo et al., 2010; Lukiw, 2012; Denzler and Stoffel, 2015; Fang and Bartel, 2015; Karnati et al., 2015). Interestingly, multiple independent studies from different laboratories whose research focuses on brain gene expression patterns indicate that in sporadic AD about 1/3 of all brain genes are up-regulated and about 2/3 of all brain genes are down-regulated (Loring et al., 2001; Colangelo et al., 2002; Lukiw, 2004; Ginsberg et al., 2012). The generalized up-regulation of the potent transcriptional activator NF-kB in AD brain was therefore somewhat perplexing since NF-kB is known as a strong inducer of inflammatory gene expression (Lukiw and Bazan, 1998; Lukiw et al., 2008; Cui et al., 2010; Devier et al., 2015; Kaur et al., 2015; Srinivasan and Lahiri, 2015). Interestingly most of the up-regulated pathogenic, pro-inflammatory genes in sporadic AD brain are directly under NF-kB transcriptional control, while down-regulated mRNAs and their expression appear to be under negative regulatory control by miRNAs which have NF-kB regulatory sites in their promoters (Figure 1; Lukiw and Bazan, 1998; Lukiw et al., 2008; Cui et al., 2010; Lukiw, 2012a,b, 2013a; Devier et al., 2015; Kaur et al., 2015; Srinivasan and Lahiri, 2015). Indeed the observed upregulation of multiple miRNAs in neurodegenerative disorders such as AD may in part explain the large number of brain gene mRNAs that are found to be consistently down-regulated in anatomical regions of the human brain targeted by the AD process (Loring et al., 2001; Colangelo et al., 2002; Lukiw, 2004; Ginsberg et al., 2012).

## Pathogenic miRNAs—up- and down-regulation or “*de novo”* appearance

It has been almost 10 years since the first reports of altered miRNA abundance and speciation: (i) in anatomical regions of the brain targeted by the AD process after post-mortem examination, (ii) in blood serum, and (iii) in cerebrospinal fluid (CSF; Lukiw, 2007; Schipper et al., 2007; Cogswell et al., 2008). Since then an in depth overview of the peer-reviewed literature has provided no general consensus of what miRNAs are up-or-down regulated in any tissue or biofluid compartment in many thousands of AD patients (Sethi and Lukiw, 2009; Hill and Lukiw, 2015; Sanei and Chen, 2015). To treat AD using an AM approach we must first be certain that our miRNA targets are really up- or down-regulated and not the consequence of some artefactual analytical technique or sample-based concern. It is clear that there are really three kinds of mature miRNA classes in human disease: (i) those that are already being expressed and their expression is down-regulated in the disease state; (ii) those that are already being expressed and their expression is upregulated in the disease state; and (iii) those whose expression appears **“*de novo*”** and their new expression correlates to the **“*onset”*** or **“*emerging presence”*** of the disease state (Sethi and Lukiw, 2009; Ramsingh et al., 2010; Gabriely et al., 2011; Hill and Lukiw, 2015; Leung, 2015; Sanei and Chen, 2015; Yuan et al., 2015). In the study of miRNAs in human post-mortem AD tissues it is probably a sensible idea to avoid the study of down-regulated miRNAs as their “***down-regulation***” may be relevant to the relatively short half-life (~1–3 h) of miRNAs in AD tissues and their **“*reduced presence”*** under pathophysiological intracellular conditions which promote the rapid degeneration of neuromolecular components (Sethi and Lukiw, 2009; Ramsingh et al., 2010; Hill and Lukiw, 2015; Sanei and Chen, 2015). The study of **“*up-regulated miRNAs”*** in AD is probably a better choice since if these miRNAs are still up-regulated in the intensively degradative cellular environment of AD tissues, then their up-regulation is probably real and non-artefactual (Sethi and Lukiw, 2009; Ramsingh et al., 2010; Hill and Lukiw, 2015; Sanei and Chen, 2015). No miRNA has yet been identified in AD that appears “***de novo”*** with the initiation or onset of the disease, in contrast to certain cancer-associated miRNAs such as miRNA-10b or miRNA-33 that appear to be previously silent or quiescent and subsequently are **“*super-activated,”*** i.e., up-regulated from zero-abundance, such as is seen in malignant glioblastoma brain tumors and the onset of gliomagenesis [(Gabriely et al., 2011; Teplyuk et al., 2012; unpublished data); AM Krichevsky, personal communication]. The abundance of miRNAs measured in brain biopsies, in blood serum or in the CSF of AD patients, which can be obtained in 1 h or less, are not as prone to this post-mortem related miRNA instability problem, and may be the method of choice when ascertaining actually what miRNA species are dysregulated over the initiation and course of the AD process. Importantly, because of human genetic individuality the profile of these up- and/or down-regulated miRNAs appear to differ between one AD patient and the next, however a “***personalized miRNA profile***” may be a characteristic feature for each individual AD patient and thus serve as a blueprint for AM-based therapeutic strategies (see below).

## Throttle up and throttle down—seed sequences and variable inhibition

In higher eukaryotes the recognition of the mRNA's non-coding 3′untranslated region (3′UTR) by miRNA via base pair complementarity is modulated by an essential “***seed sequence***” essential for the binding of the mature miRNA to the target mRNA. This seed sequence is typically a conserved heptameric ribonucleotide sequence most often situated at positions 2 through 7 from the miRNA's 5′-end (Bartel, 2009; Denzler and Stoffel, 2015; Fang and Bartel, 2015; Karnati et al., 2015). Although base pairing of a miRNA and its target mRNA 3′UTR do not perfectly match over the ~21–25 nt of the unique miRNA sequence, the “seed sequence” is always perfectly complementary (Sethi and Lukiw, 2009; Blennow et al., 2015; Hill and Lukiw, 2015; Sanei and Chen, 2015). Along the same lines of reasoning it may be possible to use AM sequences of variable complementarity to their up-regulated miRNA targets to achieve a “throttling down effect” on their expression and ultimate physiological effects to achieve the desired therapeutic endpoint. Put another way the ribonucleotide sequence of the AM may be engineered to not completely match that of its target miRNA with the goal of not totally quenching the expression of its target miRNA but rather only to down-regulate its abundance to achieve a desired therapeutic outcome. The situation would be similar for bona-fide down-regulated miRNAs in that protected miRNAs with extended stabilities may be added to the system to achieve the desired therapeutic result. Tailoring the dose and stability of the AM (or exogenously added stabilized miRNA) may also be of use in obtaining the most efficacious and sought after pharmacological effect (Sethi and Lukiw, 2009; Monteys et al., 2014; Baumgart et al., 2015; Blennow et al., 2015; Hill and Lukiw, 2015; Sanei and Chen, 2015) (see below).

## NF-kB inhibitors, anti-miRNA (AM) delivery systems and pathogenic gene expression

A considerable amount of independent laboratory research data recently generated has come to the same conclusion that the pro-inflammatory transcription factor NF-kB (p50/p65 dimer) is a powerful disruptor of normal homeostatic gene expression functions in the AD brain, and other progressive age-related neurodegenerative diseases. These include the significant enhancement in the transcription of certain brain miRNAs and the inducible up-regulation of their expression by NF-kB (Lukiw and Bazan, 1998; Lukiw et al., 2008; Cui et al., 2010; Lukiw, 2012a,b, 2013a; Devier et al., 2015; Kaur et al., 2015; Srinivasan and Lahiri, 2015). Interestingly, while NF-kB regulates the expression of multiple miRNAs, the expression and activity of NF-kB can be directly or indirectly up-regulated or down-regulated by various miRNAs; the reciprocal regulation between miRNAs and NF-kB existing in the form of multiple positive and negative feedback loops in various disease states has been recently reviewed in depth (Yuan et al., 2015). Because a particularly pathogenic set of AD-relevant pro-inflammatory miRNAs appear to be under NF-kB-mediated transcriptional control it seems reasonable to speculate that in addition to AM strategies, NF-kB inhibitors may play some future role in novel therapeutic strategies to address neurodegenerative diseases in general and to AD in particular (Lukiw et al., 2008; Cui et al., 2010; Lukiw, 2012a,b, 2013a; Devier et al., 2015; Kaur et al., 2015; Srinivasan and Lahiri, 2015).

While the use of NF-kB inhibitor(s) and AM directed strategies represent an obvious therapeutic choice in the future clinical management of AD and related neurological disorders for which miRNAs are now known to play critical pathological roles, the delivery systems used to administer these drugs to the AD patient are often complicated and problematic. For example, suitable protective drug carriers containing NF-kB-inhibitory and/or AM cargos, perhaps in combination with other drugs, may be required and these kinds of **“*drug cocktail”*** strategies are being widely researched for optimization of effective drug delivery in cancer therapeutics (Janssen et al., 2013; Lau et al., 2014; Gui et al., 2015; Kang et al., 2015; Properzi et al., 2015; Shi, 2015; Srivastava et al., 2015).

Chemical modifications of miRNAs including their use as locked nucleic acids (LNAs) significantly prolongs miRNA half-life and stability both *in vitro* and *in vivo* (Bartel, 2009; Lukiw, 2012; Monteys et al., 2014; Denzler and Stoffel, 2015; Fang and Bartel, 2015; Karnati et al., 2015). In a related application, LNAs have been used to synthesize the first miRNA-targeted drug Miravirsen, an inhibitor of the liver-specific miRNA-122 normally essential to hepatitis C virus (HCV) replication (Janssen et al., 2013). In the last 2 years the use of small, naturally occurring or engineered membrane-enveloped nanovesicles called exosomes represent a novel approach to drug delivery and disease management (Kang et al., 2015; Properzi et al., 2015; Shi, 2015; Srivastava et al., 2015). Several excellent recent reviews are available on LNA-based, nanovesicle and related miRNA delivery systems and the interested reader is encouraged to access and reference these recently compiled volumes (Janssen et al., 2013; Gui et al., 2015; Kang et al., 2015; Properzi et al., 2015; Shi, 2015; Srivastava et al., 2015; van der Pol et al., 2016).

## The heterogeneous nature of miRNA expression in AD brain

Lastly, sporadic AD involves the progressive age-related mis-regulation of numerous neurobiological pathways at multiple molecular, genetic, epigenetic, behavioral, cognitive, mnemonic, neurochemical, and neurophysiological stages. If advanced next generation RNA-sequencing and/or high density microfluidic array-based profiles of AD tissues or biofluid samples are any indication of AD variability then there are real and significant differences in AD onset, incidence, epidemiology, disease course, severity, and progression amongst different human populations (Guerreiro et al., 2012; Lukiw, 2013b; Qiu et al., 2014; Walhovd et al., 2014; Barnes et al., 2015; Blennow et al., 2015; Verhülsdonk et al., 2015; Wang et al., 2015; Zhao et al., 2015; Roth et al., 2016). It is therefore unlikely that any single miRNA in the CSF, blood serum or any other biofluid compartments from multiple human populations will represent an equal therapeutic target for every case of AD, and it would be equally unlikely that any single AM strategy would be applicable to the clinical management of every AD patient. Indeed, the data continues to accumulate that not just a single miRNA, but rather small families of pathogenic miRNAs, are probably involved in the multiple neurobiological, neurochemical and neurogenetic defects that characterize the AD process, and different miRNA families may be co-localized to different regions of brain tissue, exosome, blood serum, or CSF compartments (Lau et al., 2014; Gui et al., 2015; Hill et al., 2015a,b; Shi, 2015; Srivastava et al., 2015; Wu et al., 2015; van Harten et al., 2015). What might be particularly advantageous, however, for significantly improved future sporadic AD therapeutics would be a highly interactive, “***personalized medicine”*** approach. This would involve a comprehensive evaluation of multiple AD deficiencies including, prominently, miRNA- and mRNA-based gene expression alterations, AD-relevant DNA mutations, pro-inflammatory biomarkers, and amyloid-peptide load in the CSF, blood serum and other biofluids, combined with data from MRI- and PET-based brain imaging, and familial and clinical history and lifestyle factors that together would be extremely useful in the improvement of directed therapeutic strategies (Lau et al., 2014; Walhovd et al., 2014; Baumgart et al., 2015; Blennow et al., 2015; Gui et al., 2015; Hill et al., 2015a,b; Østergaard et al., 2015; van Harten et al., 2015; Verhülsdonk et al., 2015; Wang et al., 2015; Wu et al., 2015). Anti-miRNA (AM) therapeutic approaches seem particularly attractive since alteration in miRNA abundance appears to occur at relative early **“*initiator”*** or **“*propagatory”*** stages of the AD process while NF-kB inhibition may have too many unwanted off-target effects (Figure 1). In order to address and implement a successful AM- or NF-kB inhibitor-based treatment for AD **“*highly individualistic”*** or **“*personalized”*** treatment strategies may be required to more effectively address the specific miRNA deficits of each AD patient, including combinatorial and/or tailored AM strategies that will take dedicated care and customization to ensure a successful clinical outcome.

## Author contributions

YZ, PA, and WL collaborated interactively in the synthesis of the material presented in this Frontiers “Perspectives” article; WL wrote the article; YZ and WL have published in excess of 45 peer-reviewed manuscripts into this research area; YZ, PA, and WL declare that the research in this article was conducted in the absence of any commercial or financial relationships that could be construed as a potential conflict of interest.

### Conflict of interest statement

The authors declare that the research was conducted in the absence of any commercial or financial relationships that could be construed as a potential conflict of interest.
